# The ‘Feline Five’: An exploration of personality in pet cats (*Felis catus*)

**DOI:** 10.1371/journal.pone.0183455

**Published:** 2017-08-23

**Authors:** Carla A. Litchfield, Gillian Quinton, Hayley Tindle, Belinda Chiera, K. Heidy Kikillus, Philip Roetman

**Affiliations:** 1 Centre for Social Change, School of Psychology, Social Work & Social Policy, University of South Australia, Adelaide, South Australia, Australia; 2 Discovery Circle, School of Natural and Built Environments, University of South Australia, Adelaide, South Australia, Australia; 3 School of Information Technology and Mathematical Sciences, University of South Australia, Adelaide, South Australia, Australia; 4 Centre for Biodiversity and Restoration Ecology, Victoria University of Wellington, Wellington, New Zealand; Auburn University, UNITED STATES

## Abstract

The idea of animals possessing personalities was once dismissed by the scientific community, but has since gained traction with evidence for potential application to improve captive animal management and welfare. Although domestic cats are popular companion animals, research has tended to overlook the value of personality assessment for management and care of pet cats. The aim of this study was to investigate personality in a large sample of pet cats with a view to understanding practical implications for pet cats in the home. Personality of 2,802 pet cats, from South Australia and New Zealand, was rated by their owners utilising a survey measuring 52 personality traits. Five reliable personality factors were found using principal axis factor analysis: Neuroticism, Extraversion, Dominance, Impulsiveness and Agreeableness. Implications for the ‘Feline Five’ are discussed in relation to their potential application to improving the management and welfare of pet cats. Highly Impulsive cats for example, may be reacting to something stressful in their environment, whereas cats with low Agreeableness scores, showing irritability may indicate underlying pain or illness. Thus, the need for a systematic and holistic approach to personality that includes both the individual pet cat and its environment is recommended, and opens the door to future interdisciplinary intervention.

## Introduction

Domestic cats have been companion animals for thousands of years [1], and are popular pets in Australia, New Zealand, Europe, North America and elsewhere [2]. Yet, we know little about typical pet cat behaviour, with most behavioural studies conducted in laboratories, shelters or on free-ranging feral cat colonies [3]. This gap in knowledge is problematic since the typical environment for domestic cats is arguably the home, with tens of millions of pet cats, some kept exclusively indoors [3]. Cat owners, veterinarians, animal behaviourists and scientists often focus on the behavioural problems of stressed cats rather than on the behaviour of psychologically healthy cats and their inter- and intra-species interactions. Development of an accurate standardised ethogram (inventory of species-specific behaviours) for pet cats would facilitate creation of standards for optimal housing and welfare, like the Five Freedoms for captive animals [4].

It is possible for cats living in a multi-cat household to time-share favourite places [5], choose to spend time in close proximity with another cat [6], or play with a dog companion, with both species able to interpret each other’s behaviour correctly if they have been together from a young age [7]. Cats are more sociable than many people realise [8], and a basic understanding of cat behaviour [9] and signals (e.g. vocalisations) can allow owners to assess social stress in their cats [8], with veterinarians and other professionals able to provide this information to kitten or cat owners [10]. Understanding social interactions between cats (in the same household or neighbourhood) and between cats and their owners is important since many urban pet cats may be suffering chronic stress, as a result of lack of control over their environment [8]. A better understanding of cat personality by means of assessment could help owners improve conditions for their cats at home, thereby supporting the optimal wellbeing of their feline companions.

### Personality in animals

Personality refers to consistent individual differences in behavioural patterns [11] and is sometimes labelled as temperament [12] or behavioural syndromes [13], although a standardised term, personality, should be applicable in all cases. Personality in animals has been investigated by scientists in various fields [14], with a bias towards species considered most useful to humans, such as primates for their genetic closeness [15] and canids for their working ability [16]. However, as popular pets, an understanding of domestic cat personality could improve domestic cat welfare, by allowing carers to tailor management strategies to suit individual cats, since animal personality has been shown to influence behaviour [17], health outcomes [18], wellbeing [19], and welfare [20]. In Australia, research has tended to focus on behavioural problems related to owned and un-owned/stray cats [21, 22] rather than on the potential value of personality assessment. In Australia, almost 53,000 cats were received by RSPCA shelters in 2014–2015, with about a third of these cats eventually euthanized [23], and in the United States, an estimated 3.4 million cats enter animal shelters annually, with about 41% of these euthanized [24]. Personality assessment may increase compatibility of cat-owner placements through shelter adoption [2], with the understanding that personality of owners also influences cat behaviour [25] and therefore ideally both personality of prospective owner and cat would be assessed for compatibility [26]. For example, people scoring high on Neuroticism may have fewer and less complex interactions with their cats [25]. Cat owners through use of informed management practices, such as appropriate housing, enrichment, grouping, health and wellbeing strategies related to individual cat personalities may enhance the quality of life of their cats.

### Measures of cat personality

Early cat personality studies relied on systematic observation of cat behaviour and coding methods, which involves generating an ethogram, and then systematically recording frequency or duration of behavioural categories [16, 27, 28], such as a cat’s responses to presentations of novel objects [29, 30] or unfamiliar persons [2, 31, 32]. Though coding was considered to be objective [14], the subjective rating of comprehensive personality traits by people (usually carers) who know the animals well (the rating method), is now used more frequently [8, 25], and is considered a more reliable, practical and time-efficient approach [33]. Following the generation of a comprehensive list of species-relevant behavioural traits [34], rating usually occurs along a Likert scale to indicate the level of trait expression generally demonstrated by the animals [16]. Data are then typically reduced into a consolidated number of personality dimensions or factors, each comprising reflective personality traits using dimension reduction statistics, such as principal components analysis [28, 35].

### Research on personality in domestic cats

**Fig 1:**
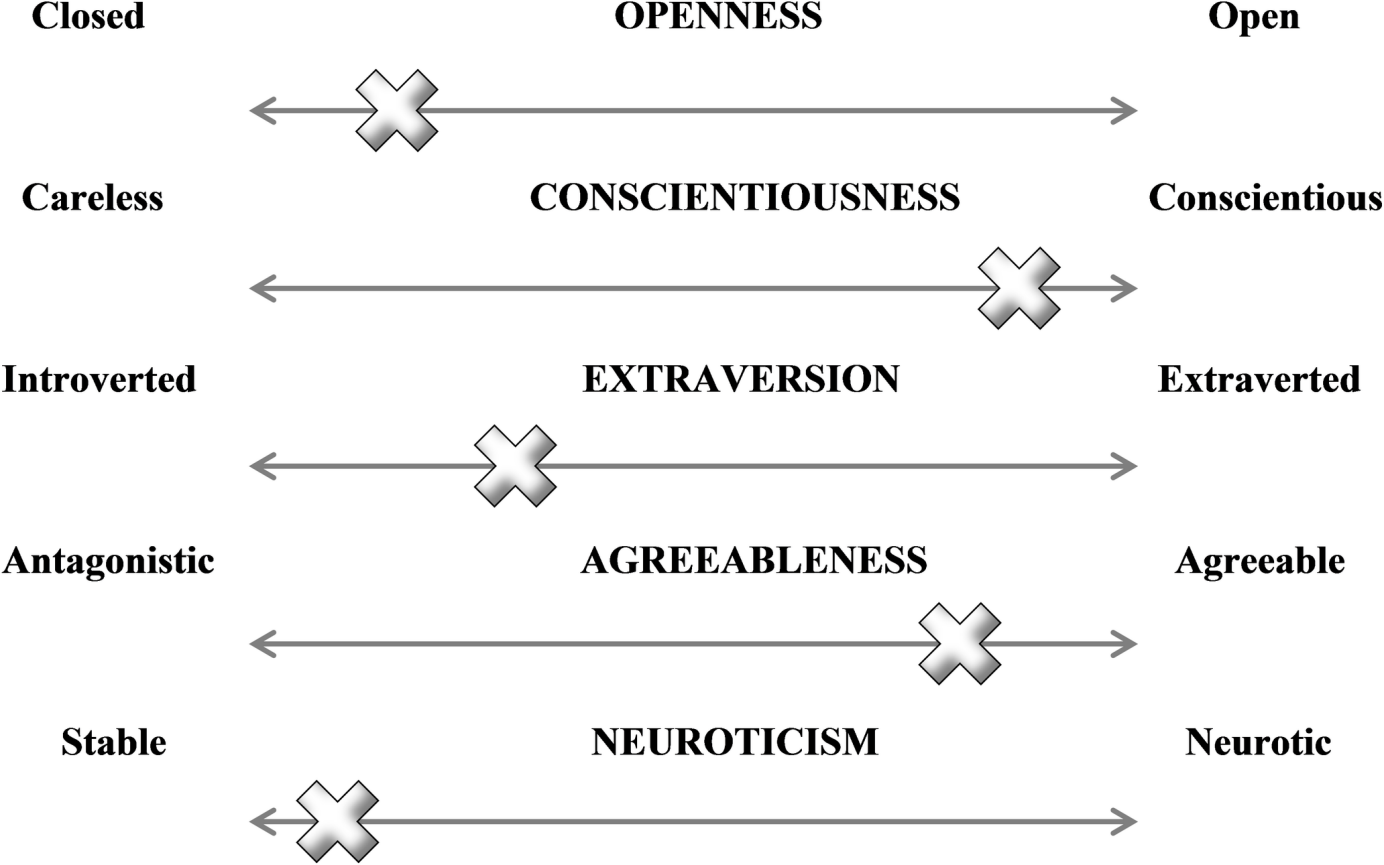
The FFM continuum and an example of how an individual's personality might be scored (based on terminology from John & Srivastava, 1999).

Investigations of cat personality have focused on either: the continuum of one personality dimension, such as dominance-warmth [36], which may allow for a more thorough investigation; or more commonly on multiple dimensions at once [37, 38], typically adapting the commonly used approach in human personality research, the Five-Factor Model (FFM), sometimes known as the Big Five [37, 39]. The FFM model is comprised of the dimensions Neuroticism, Extraversion, Openness to Experience, Conscientiousness and Agreeableness. The theory behind the model suggests an individual’s personality is determined by where they exist along each factor continuum [34] (see Fig 1 adapted from [40]).

Domestic cat personality research has tended to explore development of behaviour directed at humans, socialisation, and the shy-bold continuum of cat personality in relation to humans [30–32, 35, 41]. Individual differences exist in the extent to which cats accept the approach of, or handling by an unfamiliar person [32, 35], which is potentially influenced by the friendliness of a cat’s sire as well as socialisation effects [30, 41]. The shy-bold continuum in relation to inter-cat social behaviour has also been investigated [42], with bolder cats more vulnerable to feline immunodeficiency virus, which has potential health implications for pet cats in the home.

### Methodological challenges facing studies of cat personality

A multidimensional examination of personality in cats [43] served as a foundational study design, which used subjective trait ratings in a personality survey designed for rhesus monkeys (*Macaca mulatta*), which was modified following behavioural observation of cats in a laboratory over three months. Despite the limited generalizability of the sample (*n* = 14, female only, and laboratory environment), inter-cat social behaviour was observed and recorded, allowing successful validation of initial subjective assessments. Some of the methodological limitations or weaknesses of domestic cat personality studies conducted to date include: (1) lack of information on length of familiarity between cats and the people rating them [41]; (2) lack of acknowledgement of possible influence that researchers may have on cat behaviour when observing them in their homes [25]; (3) small sample sizes, such as 14 cats [43] or 40 cats [25, 41], with 440 cats representing the largest sample rated by their owners following recruitment over the Internet [38]; and (4) insufficient reporting of reliability assessments with inter-rater/observer reliability coefficients the most commonly reported values, conveying varying levels of agreement between raters/observers in their impressions of cat personality trait expression [25, 37, 41, 43].

Another problem concerns the inconsistent nomenclature or labelling used for cat personality trait and factors. The dimension reduction technique principal components analysis (PCA), coupled with exploratory factor analysis (EFA), is recommended to analyse statistical correlations among personality traits that cluster together creating overall components [34]. For example, the highest factor loadings on traits such as anxious, insecure, tense, and so on, have resulted in labelling the factor as Neuroticism [37]. The labelling of a factor as Feeding in one study [25], with its behavioural inclination makes its applicability to personality questionable [16]. Moreover, a lack of standardised personality factor labels and their corresponding traits is obstructing replication studies and consequently progress in cat personality research [38]. Thus, a standardised use of terms guided by previous research [44], such as the well-established human FFM nomenclature is recommended.

Following identification of personality factors, researchers should assess consistency of these factors across situations [45], with evaluation of validity to determine whether the assessment tool actually measures personality [46]. For example, the two-step process [25, 43] allows subjective ratings to be converged with coded behaviours, in order to identify and validate personality factors with some respectable coefficients (e.g. > .70), thereby strengthening accuracy of findings. Content validity was likely compromised in one application of human personality traits to cats [38] since not all domains of cat personality may have been captured in their use of only the human FFM.

### Application of personality assessment for improved cat management and welfare

Previous research on captive wild felids has led to suggestions of potential application of personality assessment to captive animal management and welfare, such as socially compatible enclosure groupings created for Scottish wildcats (*Felis silvestris*) [19] and provision of ample hiding places for highly tense-fearful cheetahs (*Acinonyx jubatus*) [20]. These applications may also be relevant to domestic pet cat management, particularly for grouping of compatible cats in multi-cat households, or providing neurotic pet cats with additional hiding places. To facilitate the use of personality assessment as a means of improving pet cat management and welfare, the methodological challenges facing research in this area, including small sample sizes, must be addressed.

### Research aims

As an exploratory study, this study aimed to analyse personality in a large sample of pet cats, utilising a personality inventory completed by cat owners about their cats. The study followed on from previous research on felid personality, which has typically found between three [37] and five factors [38], with the personality dimensions Sociable, Dominant and Curious emerging with high validity across felid species [46]. The research question that this study sought to answer was: how many reliable and interpretable factors depict personality in pet cats and what traits do they represent?

## Materials and methods

### Ethics statement and study subjects

The study was conducted in New Zealand and South Australia. The methods and materials used in this study were approved by the University of South Australia’s Human Research Ethics Committee (#33220) and the Victoria University of Wellington Human Research Ethics Committee (#21064) with all cat owners consenting to participate in the study. All issues related to participant recruitment and consent were approved by Human Research Ethics Committees. Data were collected through an online survey instrument. Participants voluntarily self-selected to complete the survey and they were provided with information regarding the project and consent at the beginning of the online survey. It was a requirement for participants to confirm that they consented to being involved in the project and that they met inclusion criteria (being at least 16 years old and currently living in South Australia, for the Australian survey; being at least 18 years old and currently living in New Zealand, for the New Zealand survey) before being able to undertake the survey. As an example, a copy of the South Australian participant information for the online survey is provided (S1 Appendix).

The Human Research Ethics Committee approved 16 years and older as a participation criterion for the South Australian research as 16 year olds were able to register cats in some local council areas in South Australia and it was considered important to understand their attitudes to cat management. The Human Research Ethics Committee agreed that a 16 year old could reasonably decide to participate in the social survey and answer the questions.

Subjects included 2,802 domestic cats of varying breeds from private homes in South Australia (SA; *n* = 1,687) and New Zealand (NZ; *n* = 1,115). This included 1,377 male, 1,387 female and 38 cats of unknown sex, ranging in age from 1–20 years, with a median age of 5 years. Cats under one year of age were excluded and only surveys where respondents had answered all personality items needed for analyses were included.

### Design, measures and materials

The current study utilised a 52-item (variable/trait) survey based on a recent comprehensive Scottish wildcat personality survey [19], which pooled items from previous personality assessments on felids [20, 43, 47] and chimpanzees (*Pan troglodytes*) [48]. Minor changes to wording were made in our survey to minimise confusion and increase applicability to pet cats. ‘Friendly to conspecifics’, ‘aggressive to conspecifics’ and ‘stingy’ were replaced with ‘friendly to other cats’, ‘aggressive to other cats’ and ‘greedy’, respectively. The survey included specific definitions alongside each item, such as insecure (‘seems scared easily, jumpy and fearful in general’) to ensure a uniform understanding of the terms among participants, who were asked to rate the extent to which their cat demonstrated each personality item/trait along a seven-point Likert scale ranging from ‘not at all’ to ‘very much so’ (S1 Appendix).

This personality survey was part of a larger online survey (using SurveyMonkey [49]) created for a citizen science project called Cat Tracker, a collaborative initiative to further the understanding of domestic cats and community views on their ownership and management. Cat Tracker also tracked pet cats utilising small Global Positioning System (GPS) tracking units. The results of the larger study will be used to inform cat management. Only the cat personality component of the online survey is provided here for analysis. The other parts of the survey are not included, nor are details about the tracking of cat movement, as they are relevant to other parts of the larger study. For reference, the larger survey included sections related to cat ownership, attachment to cats, participant demographics (such as age and sex), general opinions on cats, and cat stories (provided by the participant). To be involved in the larger survey, individuals could be either cat owners or non-cat owners (although many questions including the cat personality questions, the focus of this study, were only asked of cat owners). Participants were recruited through an open invitation to participate in the project, which was promoted by project leaders in SA and NZ through partner organisations (including academic, government and animal-focused organisations), social, and traditional media. Although the project was promoted in multiple ways (including e-newsletters to organisational mailing lists), no participants were recruited directly. The media release that was used in SA is provided as an example of promotional material (S2 Appendix). In addition, the project’s popularity saw it promoted by other individuals, companies, organisations, government departments, and media outlets in various formats and locations (including those outside the study areas). The study areas of SA and NZ were selected for convenience and due to funding conditions. Project funders were based in SA and NZ, which saw research limited to these locations. The survey was open in SA from February 2015 until September 2016 (a total of 20 months), whilst in NZ data collection began at the end of 2014 and continued until the end of 2015 (a total of 12 months). Although it was suggested that participants completed the survey online, it was also available in paper format and was posted (reply paid) to potential participants upon request. Any completed paper-based surveys received by the research team were transcribed into the online survey instrument.

### Procedure

Survey data from both SA and NZ (*n* = 4,842) were downloaded from SurveyMonkey [49] and initial data screening was conducted in Microsoft Excel. Many survey respondents had not answered all of the cat personality items (*n* = 1,780) as they did not have a cat, had a cat but decided not to complete the (optional) cat personality section, or had neglected to answer some of the items. Data from those respondents were removed. The survey responses were also checked for unengaged participants (no variation in their responses to the 52 cat personality items as indicated by standard deviations of < .50), which resulted in two being excluded from analysis. The remaining sample (*n* = 3,060) was analysed in IBM SPSS Statistics V21.0. A filter was applied to conservatively exclude cats less than one year old (*n* = 258), as personalities may still be developing prior to a minimum age of four months [35]. The relationship between the length of the personality survey and the number of missed responses to survey items was investigated using Pearson’s correlation coefficient. The last item ‘eccentric’ was not included as it was found to be an outlier, with 132 missed responses.

Initial exploratory factor analysis (EFA) utilising principal axis factoring was conducted on two independent samples of personality ratings in pet cats from SA (*n* = 1,687) and NZ (*n* = 1,115). Initial results (Table 1) show the orthogonally (varimax) rotated four-factor solutions, initial and rotated eigenvalues, and percentages of variance revealing moderate-high internal consistencies, and highly similar factor extractions between both data sets. For SA, the items that clustered on factor 1 represent Neuroticism; factor 2, Dominance; factor 3, Extraversion; factor 4, Impulsiveness, and cumulatively explain an initial variance of 41.58%. A similar factor structure was revealed for NZ, cumulatively explaining an initial variance of 41.35%, but the items on factor 3 represent Self-control and factor 4, Extraversion; however, factors 3 and 4 for NZ were presented adjacently to factors 3 and 4 for SA to enable meaningful comparison (Table 1). The high similarity between factor structures cross-validated the results [50], thus justifying further analysis using a combined sample (*n* = 2,802).

**Table 1:** Cross-validation of the cat personality measurement scale using two independent samples: South Australia (SA; *n* = 1,687) and New Zealand (NZ; *n* = 1,115).

*Rotated Principal Axis Factor Analysis Loadings*								
Item	Neuroticism		Dominance		Extraversion		Impulsiveness; Self-Control	
	SA	NZ	SA	NZ	SA	NZ	SA	NZ
Insecure	**.78**	**.77**	-.03	-.05	-.04	.06	.20	-.22
Fearful of people	**.77**	**.77**	.05	.05	-.08	-.02	-.08	.03
Suspicious	**.76**	**.78**	.12	.04	-.11	-.05	-.12	.13
Anxious	**.75**	**.74**	.03	.02	-.07	.02	.22	-.23
Trusting	**-.74**	**-.77**	-.16	-.04	.18	.09	.11	-.05
Shy	**.72**	**.71**	-.04	-.07	-.17	-.15	.08	-.05
Calm	**-.66**	**-.66**	-.15	-.10	-.06	-.12	-.17	.16
Stable	**-.62**	**-.62**	-.19	-.14	-.02	-.16	**-.30**	**.31**
Friendly to people	**-.59**	**-.64**	-.22	-.13	.21	.19	.17	-.09
Tense	**.56**	**.57**	.15	.17	-.05	.03	.15	-.21
Self-assured	**-.51**	**-.51**	.07	.11	.25	.16	**-.43**	**.50**
Bold	**-.48**	**-.53**	.27	.24	**.40**	**.33**	-.11	.25
Fearful of other cats	**.46**	**.44**	-.09	-.13	-.10	-.05	.08	-.20
Cool	**-.42**	**-.40**	-.00	.02	-.08	-.16	-.22	.22
Excitable	**.36**	.29	.14	.19	**.33**	**.38**	**.31**	-.22
Solitary	**.34**	.**39**	.27	.17	-.26	-.18	-.06	.04
Bullying	-.06	-.09	**.69**	**.76**	.09	.04	-.00	.07
Dominant	-.21	-.19	**.66**	**.69**	.10	.09	-.12	.19
Aggressive to other cats	.02	.04	**.65**	**.66**	.03	-.02	-.05	.07
Defiant	-.05	-.08	**.64**	**.67**	.21	.14	-.02	.05
Irritable	.22	.17	**.61**	**.62**	-.02	.01	.10	-.06
Gentle	**-.30**	-.25	**-.58**	**-.59**	.06	-.06	-.06	.02
Aggressive to people	.19	.17	**.49**	**.50**	.00	.12	.13	-.06
Jealous	.08	.05	**.48**	**.48**	.09	.07	.08	-.04
Erratic	.24	.25	**.47**	**.47**	.19	.23	**.41**	**-.37**
Cooperative	-.18	-.18	**-.44**	**-.43**	.10	.06	-.11	.17
Submissive	.24	.20	**-.44**	**-.52**	-.03	.04	.25	-.26
Friendly to other cats	-.25	-.25	**-.41**	**-.34**	.19	.22	.14	-.06
Reckless	-.04	-.08	**.41**	**.47**	**.35**	**.37**	**.34**	-.19
Affectionate	**-.32**	**-.36**	**-.38**	**-.30**	.22	.17	.06	.04
Greedy	-.01	-.08	**.34**	**.39**	.10	.02	.19	-.15
Curious	-.22	-.18	-.04	-.06	**.69**	**.59**	-.12	**.32**
Inventive	-.19	-.14	-.03	.03	**.64**	**.52**	-.15	**.33**
Inquisitive	**-.30**	-.21	-.08	-.03	**.64**	**.59**	-.09	.28
Playful	-.19	-.15	-.23	-.16	**.58**	**.50**	.08	.11
Active	-.12	.00	-.03	-.03	**.53**	**.55**	-.19	.25
Impulsive	.05	.12	**.31**	**.31**	**.42**	**.41**	**.32**	-.21
Persevering	-.11	-.08	.16	.19	**.39**	**.33**	-.20	.26
Vigilant	.15	.23	.05	.06	**.39**	**.39**	-.27	**.30**
Constrained	.02	-.04	-.23	-.28	**-.36**	**-.38**	-.16	.15
Vocal	-.05	-.05	.01	-.01	.**31**	.23	.00	-.02
Eccentric	.11	.06	.16	.22	.29	**.31**	.22	-.16
Individualistic	.06	.06	.15	.23	.25	**.31**	-.03	.02
Aimless	.08	.07	.10	.17	-.09	.03	**.58**	**-.56**
Decisive	-.12	-.14	.08	.06	**.40**	.23	**-.55**	**.59**
Clumsy	.03	.03	-.03	.02	-.02	.00	**.52**	**-.48**
Smart	-.08	-.02	-.00	.03	**.36**	.25	**-.50**	**.59**
Deliberate	-.01	-.09	.10	-.02	.24	.10	**-.49**	**.52**
Distractible	.11	.06	.15	.17	.17	**.30**	**.47**	**-.41**
Quitting	.17	.12	.14	.13	-.14	-.04	**.30**	**-.36**
Independent	-.16	-.08	.22	.18	.03	.01	-.28	**.37**
Predictable	.03	-.03	-.22	-.27	-.18	-.24	-.27	.20
Initial eigenvalues	9.04	8.58	5.86	6.18	3.49	3.08	3.24	3.66
% of variance	17.38	16.50	11.28	11.89	6.71	7.03	6.22	5.93
Rotated eigenvalues	6.90	6.92	4.93	5.14	4.09	3.38	3.36	3.69
% of variance	13.27	13.30	9.48	9.89	7.86	6.50	6.43	7.09
Cronbach’s α	.90	.91	.85	.85	.77	.75	.72	.72

*Note*. Salient factor loadings (≥ .30) are in boldface.

Preliminary analyses involved examination of the Kaiser-Meyer-Olkin (KMO) and Bartlett’s test of sphericity to determine if the data were appropriate for factor analysis (S3 Appendix). The overall KMO measures of .92 and .91 respectively, confirmed sampling adequacy for both analyses with values exceeding the minimum level of .50 [51], and Bartlett’s Tests of Sphericity [52] were significant (*p* < .001), thus the data were considered appropriately correlated for factor analysis. The KMO values for individual items were over .72 (S3 Appendix), which is greater than the accepted level of .50 [50].

Initial analyses were run to obtain eigenvalues for each factor in the data. Ten factors had eigenvalues greater than Kaiser’s criterion of one that cumulatively explained a variance of 56.95% for SA and 57.22% for NZ. For SA and NZ as independent samples, the scree plots display the eigenvalues associated with the components in descending order against the number of the components revealed inflexion points that justified retaining four factors for each data set (S4 Appendix; [53]), whereas parallel analysis [54] justified retaining up to seven factors for SA and eight for NZ (S4 Appendix).

The factorability of the 52 cat personality survey items was also examined using several well-known criteria (S3 Appendix), and based on these preliminary analyses the 52 cat personality survey items were subjected to EFA. Exploratory factor analysis was conducted (S5 Appendix). Principal components analysis (PCA) was initially used to determine the potential number of factors within the data set, and as a result of this process four items (independent, individualistic, eccentric and vocal) were excluded (S5 Appendix). Finally, a principal axis factor analysis (PAF) was conducted on the final 48-item sample. The justifications used for each step of the preliminary and final analysis processes are outlined in detail in this section, the results section, or are provided in S3 Appendix, S4 Appendix, and S5 Appendix.

## Results

### Cat owner participants

The 2,802 cats (study subjects) were owned by 2,291 survey participants, members of the general public of SA and NZ. Participants were male (*n* = 308), female (*n =* 1,850) or did not answer (*n =* 133). About 60% of participants were aged between 21 and 50 years, with approximately 16% of participants on either side of this age range (Table 2).

**Table 2:** Descriptive statistics for participant age (*n* = 2,291).

Age range	*n*	Percentage
16–17	72	3.1%
18–20	312	13.6%
21–30	533	23.3%
31–40	457	19.9%
41–50	393	17.2%
51–60	269	11.7%
61–70	89	3.9%
71–80	19	0.8%
81+	1	0.0%
Unknown	146	6.4%

The Pearson’s correlation coefficient showed there was a strong correlation between the number of missed survey items and the length of the cat personality survey using the combined SA and NZ data, *r* = .73, *n* = 51, *p* < .01. A final PAF was conducted on the 48-item cat personality dataset with five factors cumulatively explaining a variance of 47.43%. Orthogonal (varimax) and oblique (direct oblimin) rotations were explored and resulted in similar factor extractions, as minimal correlations (< .30) were present between factors ranging from .01 to .28 (S5 Appendix). Consistent with previous research [19, 37], other recommendations [55–57] and for ease of interpretation and understanding [58], the orthogonally rotated (varimax) solutions are presented, cumulatively explaining a variance of 41.53%. All items had salient loadings over .30 and some cross-loadings, but following previous studies [19, 37, 59], items with multiple salient loadings were assigned to the factor that had the highest corresponding loading. We refer to this final five-factor solution as the Feline Five. The factor-loading matrix after rotation, the initial and rotated eigenvalues, and percentages of variance explained by the factors determining their importance are presented below (Table 3).

**Table 3:** Combined South Australian and New Zealand pet cat personality factor structures (*n* = 2,802).

*Rotated Principal Axis Factor Analysis Loadings*					
Item	Neuroticism	Extraversion	Dominance	Impulsiveness	Agreeableness
Insecure	**.81**	-.14	-.03	.06	-.03
Anxious	**.77**	-.17	.02	.07	-.06
Fearful of people	**.73**	.04	-.02	-.05	-.27
Suspicious	**.71**	.06	.02	-.09	**-.31**
Shy	**.70**	-.13	-.06	-.09	-.17
Trusting	**-.69**	.00	-.02	.07	**.36**
Calm	**-.68**	.04	-.09	-.18	.09
Stable	**-.64**	.15	-.11	-.26	.08
Tense	**.56**	-.13	.11	.12	-.14
Self-assured	**-.53**	**.46**	.13	-.12	.04
Bold	**-.52**	**.37**	.17	.28	.02
Fearful of other cats	**.46**	-.14	-.10	-.03	-.04
Cool	**-.45**	.08	.01	-.17	-.05
Excitable	**.39**	.04	.15	**.38**	.15
Decisive	-.14	**.62**	.16	-.17	.03
Smart	-.09	**.60**	.02	-.11	-.02
Curious	-.17	**.59**	-.03	.28	.29
Inventive	-.14	**.56**	.03	.22	.26
Active	-.09	**.53**	-.12	.28	.09
Inquisitive	-.21	**.53**	-.03	.27	**.32**
Vigilant	.16	**.48**	.01	.13	-.03
Deliberate	-.05	**.48**	.14	-.23	-.01
Aimless	.11	**-.45**	.06	**.37**	.06
Clumsy	.11	**-.40**	.03	.21	.22
Persevering	-.09	**.40**	.19	.11	.08
Quitting	.16	**-.31**	.07	.18	-.06
Bullying	-.04	.06	**.79**	.14	-.07
Dominant	-.19	.16	**.70**	.11	-.11
Aggressive to other cats	.04	.04	**.69**	.10	-.15
Jealous	.13	-.02	**.59**	.10	.04
Defiant	-.09	.16	**.56**	**.30**	-.19
Submissive	.25	-.19	**-.46**	.02	.17
Greedy	.02	-.11	**.45**	.15	.11
Friendly to other cats	-.21	.04	**-.37**	.10	.28
Impulsive	.07	.10	.12	**.60**	-.02
Erratic	.21	-.13	.23	**.60**	-.21
Reckless	-.04	.04	**.31**	**.55**	.02
Predictable	.05	.02	-.02	**-.48**	.13
Distractible	.13	-.18	.07	**.48**	.10
Constrained	.01	-.15	-.10	**-.47**	.01
Affectionate	-.19	.06	-.07	-.14	**.61**
Friendly to people	**-.50**	-.02	.01	.05	**.52**
Gentle	-.19	.00	**-.33**	**-.32**	**.49**
Playful	-.08	**.34**	-.09	.22	**.47**
Solitary	.26	-.09	.06	.00	**-.44**
Irritable	.12	-.04	**.39**	**.32**	**-.43**
Cooperative	-.12	.12	-.26	-.24	**.34**
Aggressive to people	.12	-.02	**.30**	**.32**	**-.33**
Initial eigenvalues	8.78	5.77	3.35	3.13	1.76
% of variance	18.24	12.03	6.97	6.51	3.67
Rotated eigenvalues	6.43	3.88	3.62	3.32	2.68
% of variance	13.40	8.08	7.55	6.92	5.58
Cronbach’s α	.90	.80	.80	.72	.78

*Note*. Salient factor loadings (≥ .30) are in boldface.

In order to choose suitable labels for each of the five personality factors, a comparison of labels used in other animal and particularly felid studies was undertaken (S6 Appendix). Thus, factor 1 represents Neuroticism, factor 2 represents Extraversion, factor 3 represents Dominance, factor 4 represents Impulsiveness, and factor 5 represents Agreeableness. The initial eigenvalues showed that the factors explained a variance of 18.24%, 12.03%, 6.97%, 6.51% and 3.67% respectively, which levels out on rotation (Table 3). The internal consistencies of each of the five factors was assessed using Cronbach’s alpha to determine their reliabilities and resulted in a high coefficient of .90 for Neuroticism and acceptable/moderate [60] coefficients of .80 for Extraversion and Dominance, and .72, and .78 for Impulsiveness and Agreeableness, respectively. No considerable increases in alpha levels could have been accomplished by further item removal.

In summary, following the removal of four of the 52 cat personality survey items, the final analysis yielded five distinct factors that depict personality in pet cats from the combined SA and NZ sample and determined the factors were moderately to highly consistent.

### Factor scores

Weighted sum factor scores were created in Microsoft Excel. The salient item loadings in each factor were multiplied by the Likert scale score for each corresponding survey item. The resulting values were then summed together to create factor scores for individual cats on all five factors [61]. These results were then provided to cat owners, who had opted to receive the results, through individual reports that illustrated their cats’ score on each of the five factors, and how this compared to other cats using a low, typical, and high format. The report also contained a brief description of what the personality results may mean in terms of application to cat management, particularly if their cat had extreme scores on any of the personality factors.

## Discussion

This study sought to determine the number of reliable and interpretable factors that depict personality in pet cats and analyse what traits the factors represent. The results suggest that there are five factors of domestic cat personality and that these represent traits related to Neuroticism, Dominance, Impulsiveness, Agreeableness, and Extraversion. This study is the first of its kind to utlilise a transnational sample of this magnitude (*n* = 2,802) and build on previous research to fill in a number of methodological gaps. The cross-validation technique analysing data from two independent samples (SA and NZ) strengthened the research providing a reliable and valid measure of pet cat personality upon which future animal personality research can build. Management and welfare implications for pet cats in the home have been largely neglected, and how this research can be used within this field is discussed subsequently. However, there are some limitations of the study which can be addressed in future research. Survey length may affect rater accuracy and engagement if they are too long [62, 63]. We found that the final item ‘eccentric’ was missed often enough to become an outlier. However, this was the only item that had an additional open-ended response option (to explain any unusual behaviour in their cat), which may have confused raters or as the final question in a list of 52 items, the survey may have been too burdensome to complete. This item is still worth including, as unusual or idiosyncratic aberrant behaviour may be an indicator of stress, but may be better placed elsewhere in the measure. The elimination of four items during statistical data reduction has the potential to address the limitation of length. Next, the majority of survey respondents were female, which is consistent with some previous research finding females typically complete surveys more often than males [64], and some argue that adult women are generally ‘predestined’ to be the main human companion in human-cat dyads [65]. Future research could aim to have a more representative sample by setting target sex/gender ratios aligned with population data. Finally, not asking raters how long they had known the cats prior to rating their personalities may also be a limitation to the validity of the measure. However, this issue is most likely to be prevalent for animals in zoos or in shelter environments, where keepers or staff may rotate frequently, or spend little time with individual animals, rather than with pets who have the opportunity to behave more naturally in their environment [46].

### Domestic pet cat personality structure: The feline five

Pet cat personality ratings from SA and NZ revealed five factors with acceptable-high internal consistency: Neuroticism, Extraversion, Dominance, Impulsiveness and Agreeableness. Based on the clusters of traits, previous research and the FFM nomenclature, the labelling of four out of the five cat personality factors was clear, namely: (1) Neuroticism- reflects strongest levels of traits, such as insecure, anxious, fearful of people, suspicious and shy; (2) Dominance- reflects bullying, dominant and aggressive to other cats; (3) Impulsiveness- reflects impulsive, erratic and reckless; and (4) Agreeableness- reflects affectionate, friendly to people and gentle. However, our fifth factor Extraversion also revealed traits normally associated with Self-control in Scottish wildcats [19] including decisive, aimless, persevering and quitting. Our decision to label the factor Extraversion (S6 Appendix) was based on the fact that traits reflecting Extraversion, including: active, vigilant, curious, inquisitive, inventive, and smart all loaded onto this factor and have also been reported for other pet cats [25], captive wild felids [20, 47, 66] and orangutans (*Pongo pygmaeus* and *Pongo abelii*) [59].

Our Feline Five factors of Extraversion, Neuroticism and Agreeableness appear generally consistent with the Big Five human personality assessments, along with the addition of Dominance in nonhuman animal personality assessments [67]. The Feline Five introduces a more comprehensive overall structure of pet domestic cat personality based on the largest sample size to date. The similarities between our factor structures and those reported in other studies of animal and domestic cat personality (S6 Appendix), suggest that some factor labels could be standardised, enabling comparison and discussion of their practical implications. Individual cats will exist somewhere along a continuum between low and high scores for each of the five factors. Since cross-species similarities in personality exist between pet cats and captive wild felids [19, 20] or other mammals [59], and personality has played an important role in the health and wellbeing outcomes of captive animals, personality is likely to have similar implications for pet cats. Being able to accurately assess personality of pet cats, and consider possible suggestions for improvements in pet cat management, may help owners manage individual cats in a way that optimizes their welfare.

### Applications of feline five personality scores to management of pet cats

The personality profiles of their cats may not only be interesting to cat owners, but may be used to improve welfare, particularly when an individual cat has unusually high or low scores on a factor. Awareness of results being considered extreme on a scale, compared to a sample of 2000+ pet cats, allows the owner to seek advice and consider changes to the environment or management of their cat. Personality profiling may be particularly useful for managing multi-cat households, ideally before obtaining a new cat. The following information provides specific examples.

Cats that score high on Neuroticism (shy) may be stressed and benefit from an assessment of social stress [8] by observing any interactions between the neurotic cat and others (human or non-human animals). These cats may benefit from additional hiding places around the home or access to quiet areas (like cheetahs with high tense-fearful scores [20]). All cats that are allowed to roam outside are at greater risk of disease transmission [42] or injury (road deaths or fights). Cats with low scores for Neuroticism (i.e., they are bold) may travel further (if not confined), compounding this risk.

Cats with high scores for Extraversion (smart, curious, inventive) may need additional stimulation and more complex environmental enrichment to avoid boredom [9], such as extra room to play, additional sensory items or toys, and social interactions with humans and/or other animals [3]. Low scores for Extraversion (clumsiness, aimlessness) may indicate age-related health issues, such as cognitive dysfunction [68], or other health problems, thus requiring further individual assessment from a veterinarian.

High scores on Impulsiveness (erratic, reckless) may also indicate a stressful environment [8, 69], with negative effects on a cat’s health and welfare [70, 71], and owners may need to seek advice from an animal behaviourist to locate the source of stress. Low scores for Impulsiveness are likely to be indicative of cats that are well adjusted to their environment and enjoy routine.

High scores for Agreeableness (friendly) are likely to represent cats that are well adjusted and ‘happy’, potentially serving as a source of enrichment for other cats (see captive elephants (*Loxodonta africana*) [72] and tigers (*Panthera tigris tigris*) [73]). Owners typically desire friendly cats [30] for their therapeutic benefits [69], and as a result friendly cats are more likely to be adopted from shelters [2]. Low scores for Agreeableness (irritable/aggressive towards people) may reflect poor socialisation [30], frustration [71], or underlying pain or illness [74].

Further research is needed to understand how extreme scores for the Feline Five factor of Dominance may be used for improving cat welfare. Most of our knowledge about sociality of domestic cats comes from behavioural observations of free-living (feral) colonies, where related adult females cooperate in raising kittens, while there is competition between adult males [8]. High scores for Dominance reflect a cat that is likely to bully other cats in the household, potentially causing stress, aggression or injury [75], with object (food) and social (inter-cat) dominance behaviours observed in situations with forced grouping of cats [29]. This particular personality factor may have a biological basis as oxytocin (a neuropeptide) has been associated with Roughness (consisting of traits irritable, dominant, forceful & moody) in cats [76].

In summary, our Feline Five factors were: Neuroticism, Extraversion, Dominance, Impulsiveness and Agreeableness. Accurate assessment of pet cat personality may help owners manage their cats in a way that optimises cat welfare. Additionally, a greater understanding of cat personality may help owners notice changes in their cat and seek professional assessment by a veterinarian and/or animal behaviour specialist [8, 17, 77].

## Supporting information

S1 Appendix Relevant survey information. (PDF)

S2 Appendix Media release for SA cat tracker project. (PDF)

S3 Appendix Preliminary analyses. (PDF)

S4 Appendix South Australia and New Zealand as independent samples. (PDF)

S5 Appendix Exploratory factor analysis. (PDF)

S6 Appendix Comparison of factor labels used in other animal and particularly felid studies compared with the current study. (PDF)
