# Supplementary material for: The ‘Feline Five’: An exploration of personality in pet cats (Felis catus)
Source: PLoS One. 2017 Aug 23;12(8):e0183455. doi: 10.1371/journal.pone.0183455 (PMC5568325; doi:10.1371/journal.pone.0183455)
Supplement: S1 Appendix — (PDF) [file pone.0183455.s001.pdf]

# S1 Appendix: Relevant Survey information

## Part A: Participant Information Sheet

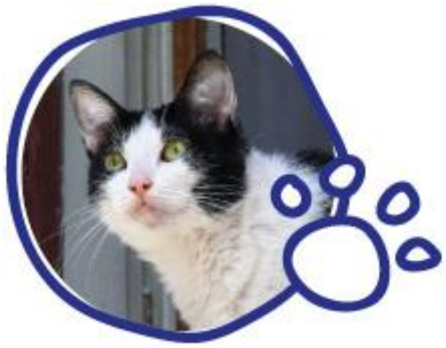

Welcome to the ***Cat Tracker*** project and thank you for taking part!

This survey can be completed by any adult (16+) in South Australia – **you can complete the survey even if you don't own a cat.**

**If you are not in South Australia:** we are hoping to take this project national during 2016-17 and track more cats if we can gain all the necessary funding and approvals. Please visit: http://bit.ly/Discovery-Circle-eNews to sign up to our newsletter to keep up to date.

**INCENTIVE:** By completing the survey you have the chance to receive one of ten **$50 Coles/Myer gift cards.**

The survey will help us to understand more about cats, their behaviours and personalities, and their relationships with their owners. If you own a cat, you may be especially interested in the results of the cat personality section (for your cat/s). You can request a copy of the results within the survey.

The survey will take approximately 25 minutes to complete, but will vary depending on cat ownership (shorter if you don't own a cat). There are options within the survey to reduce the survey length. **If you need to change your answers during the survey, please use the 'previous' button at the bottom of the survey rather than the 'back' button on your browser window.**

**Response to this survey is voluntary and your responses will be kept confidential!** This project has been approved by the University of South Australia's Human Research Ethics Committee. Click here for more information.

## Part B: Extra Ethics Information

# Cat Tracker survey

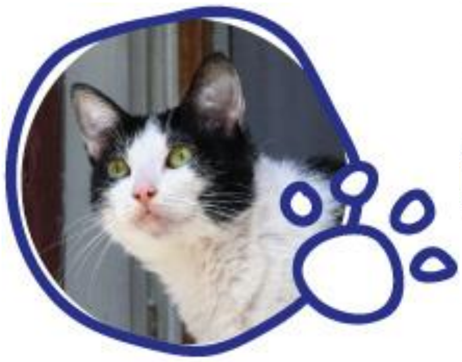

Discovery Circle

# Additional information for participants

**By completing this questionnaire you are indicating your consent to participate in the survey part of this research project**. Participants are free to withdraw from the research project at any stage without affecting their status now or in the future. Individual's responses will be kept confidential by the researchers (Dr Philip Roetman and Dr Carla Litchfield) and will not be identified in the reporting of the research.

**Incentives**: Within the survey you can choose if you would like to be included in a random draw to receive one of ten **$50 Coles/Myer gift cards** that we are giving away. All people who complete the survey and request to participate will be included in the random draw. You will need to provide your contact details if you would like to go in the random draw.

A report on the overall results of this research will be provided to participants (if requested in the survey) and reported to organisations interested in cat welfare and management – no individuals will be identified in these reports.

**This project has been approved by the University of South Australia's Human Research Ethics Committee**. If you have any ethical concerns about the project or questions about your rights as a participant, please contact the Executive Officer or this Committee, telephone +61 8 8302 3118; or email vicki.allen@unisa.edu.au.

This is an internet-based survey. Every effort will be made to ensure that responses are confidential (e.g. we are using robust and secure survey software and storing data on secure servers); however, the researchers cannot guarantee the confidentiality or anonymity of material transferred by email or the internet. Data will be stored on servers in secure facilities at the University of South Australia. It will be stored for no less than 7 years. This length of time will allow for the analysis of the data and to respond to any queries about the outcomes of the project.

If you would like to contact the researcher, Dr Philip Roetman, please call the Discovery Circle Research Office at the University of South Australia on (08) 8302 9999.

## Part C: Inclusion criteria questions

Please confirm that you are eligible to participate in this project:

(multiple choice options)

**1.** I am 16 years old or over

- Yes
- No

**2.** I currently live in South Australia

- Yes
- No

## Part D: Option to complete Cat Personality Test questions

**39.** The next section is about the personality of [cat's name]. We will use this section to report back to you on your cat's personality. However, as this section alone takes 5 minutes to complete, you have the option to skip it.

(multiple choice options)

- Do the cat personality test (takes 5 minutes)
- Skip the personality test (we won't be able to report your cat's personality to you)

# Part E: Cat Personality Test questions

This page includes questions that form a cat personality test. We can send you the results of this test if you provide your email address at the end of this survey.

**Please note that if you wish to receive a personality report for your cat, all of the personality questions must be answered.**

With regard to this cat, please rate whether he or she in general demonstrates each behaviour or personality characteristic. A definition of each personality characteristic is provided (in brackets). Some personality characteristics (or words) may differ from your idea or definition, so please use the definitions provided here.

**40.** With regard to [cat's name], please rate whether he or she in general demonstrates each behaviour or personality characteristic.

(Seven-point scale ranging from 'Not at all' to 'Very much so')

- **Vigilant** (watchful or observant; spends a lot of time attending to his/her surroundings)
- **Stable** (reacts to his/her environment in a calm way)
- **Bold** (daring, not restrained or tentative, doesn't hesitate)
- **Clumsy** (relatively awkward or uncoordinated during movements, e.g. when walking, climbing, or playing)
- **Defiant** (assertive or difficult or challenges the usual dominance order with other cats or people in your household, even if there are unfavourable consequences for this cat)
- **Gentle** (responds to others in an easy-going manner, which is not rough or threatening)
- **Constrained** (controlled and not very impulsive)
- **Inquisitive** (he/she seems drawn to new situations, objects, or animals and behaves as if he/she wishes to learn more about others or objects within view)
- **Inventive** (more likely than other cats to do new things including novel or problem-solving behaviours, e.g. opening a cupboard or entering bags or boxes)
- **Irritable** (often seems in a bad mood, or is impatient and easily provoked to anger or other agonistic or threatening/aggressive behaviour)
- **Distractible** (easily distracted and has a short attention span)
- **Erratic** (Inconsistent or widely varying in behaviour and moods)
- **Solitary** (spends time alone or avoids company by choice)
- **Impulsive** (displays spontaneous or sudden behaviour that was not anticipated)
- **Quitting** (readily stops or gives up activities that have recently been started)
- **Independent** (behaviour not influenced or controlled by other animals, events, or things)
- **Smart** (quick and accurate in judging and comprehending situations; learns quickly to associate events and appears to remember for a long time)

- **Jealous** (often troubled by other cats, pets or people who are in a desirable or advantageous situation such as having food, a choice location, or access to social situations. May attempt to disrupt activities of advantaged cats)
- **Fearful of other cats** (retreats readily or moves away from other cats)
- **Persevering** (continues in a course of action, task, or strategy for a long time or continues despite opposition)
- **Greedy** (excessively desirous or covetous of food, favoured locations, or other resources and unwilling to share these resources with other cats/pets)
- **Friendly to other cats** (initiates proximity with or getting close to other cats; approaches other cats readily and in a friendly manner, e.g. purrs, rubs)
- **Submissive** (often gives in or yields to another cat/pet)
- **Dominant** (controlling, exerting forcefulness, powerful with respect to other cats/pets)
- **Reckless** (rash or unconcerned about the consequences of his/her behaviours)
- **Predictable** (consistent and steady behaviour over extended periods of time; sticks to a behavioural routine or set of activities)

**41.** With regard to [cat's name], please rate whether he or she in general demonstrates each behaviour or personality characteristic.

(Seven-point scale ranging from 'Not at all' to 'Very much so')

- **Suspicious** (not trusting; does not approach easily, e.g. human or animal visitors in the house)
- **Individualistic** (behaviour stands out or is unusual/different compared to that of other cats)
- **Affectionate** (warm attachment to or closeness with other cats, pets or people, e.g. grooming, touching, or lying next to other cats, pets or people)
- **Insecure** (seems scared easily, jumpy and fearful in general)
- **Bullying** (overbearing and intimidating towards other cats)
- **Curious** (seeks out or investigates novel situations)
- **Aimless** (seems to behave without any clear purpose or direction)
- **Deliberate** (seems to behave in an intentional or planned way)
- **Tense** (shows restraint in movement and posture, e.g. almost frozen in position)
- **Fearful of people** (retreats readily or moves away from people)
- **Cool** (unaffected by emotions and usually undisturbed, assured, and calm)
- **Aggressive to people** (reacts in a hostile way or attempts to attack/threaten people)
- **Calm** (Not easily disturbed by changes in the environment)
- **Aggressive to other cats** (reacts in a hostile way or attempts to attack/threaten other cats)
- **Excitable** (overreacts to changes in the environment)

- **Friendly to people** (initiates proximity or closeness to people by approaching readily and in a friendly manner, e.g. purring and/or rubbing against legs)
- **Playful** (initiates and engages in non-aggressive play behaviour with objects, which may seem meaningless)
- **Vocal** (frequently and readily vocalises)
- **Decisive** (seems determined and purposeful in his/her activities)
- **Self-assured** (moves in a seemingly confident, well-coordinated, and relaxed manner)
- **Anxious** (interested but fearful and uneasy; seems to change his/her mind about approach or withdrawal)
- **Trusting** (not suspicious and approaches easily, e.g. human or animal visitors in the house)
- **Active** (moves frequently, e.g. often walks, runs, stalks)
- **Cooperative** (is compliant; willingly behaves when asked to do something)
- **Shy** (reluctant to approach other animals, novel objects or new situations)
- **Eccentric** (shows unusual behaviours,) If your cat seems eccentric, please describe the behaviour [open-ended response]

## Part F: Human respondent demographic questions

**84.** What is your gender?

(multiple choice)

- Male
- Female

**85.** What is your age?

(single choice, drop-down box response)

- 16-17
- 18-20
- 21-30
- 31-40
- 41-50
- 51-60
- 61-70
- 71-80
- 81+
