# Supplementary material for: The ‘Feline Five’: An exploration of personality in pet cats (Felis catus)
Source: PLoS One. 2017 Aug 23;12(8):e0183455. doi: 10.1371/journal.pone.0183455 (PMC5568325; doi:10.1371/journal.pone.0183455)
Supplement: S2 Appendix — (PDF) [file pone.0183455.s002.pdf]

S2 Appendix: Media Release for SA Cat Tracker Project

Cat Tracker: Discover the secret life of your cat

February 24 2015

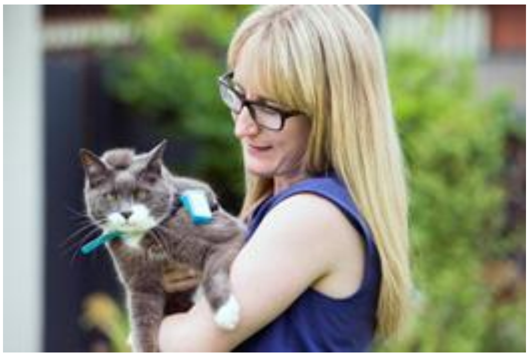

Ever wondered where your cat ventures to? Now's your chance to find out, by joining in with the University of South Australia's new Cat Tracker project.

The project is being led by Dr Philip Roetman from the Discovery Circle, UniSA's Citizen Science initiative and is designed for cat owners and school classes to get involved.

"We'll provide GPS tracking devices for cat owners in South Australia. They are easy-to-use, and will help people discover the places their cats visit," Dr Roetman says.

"People who want to participate will fill out an online survey first, which includes a cat personality test. Then, if they would like to track their cats, we'll send out the GPS tracker. Once they have completed the tracking, we will give them a report on their cat's personality and where their cat goes."

Cat Tracker will build upon a similar initiative, which is being undertaken in North Carolina, USA, and is yielding some interesting results.

"The team in North Carolina are finding that urban cats don't travel very far and that they usually stick to the built environment. I'm fascinated to see if these preliminary findings are backed-up as more cats are tracked, and if cats behave in the same way in Australia," Dr Roetman adds.

"Previous research has been done on too few cats to make any generalisations. By collaborating with the community, we hope to track 500 cats in South Australia and get a better understanding of cat behaviour here."

Dr Carla Litchfield, an expert on animal behaviour and project collaborator, explained that the outcome of the research would help owners understand more about their pets.

"Anyone who has had more than one cat knows that each cat has its own personality and this study will help us understand different cat personalities. Understanding a cat's personality can help to care for it. It will also be interesting to see whether cats who demonstrate bolder personality traits travel further afield compared to cats which may be shy in nature" Dr Litchfield says.

The Discovery Circle team have also developed extensive resources for school teachers to use with classes from Reception to Year 9. The resources are aligned with the Australian curriculum, with links to learning areas including science, mathematics, geography, history and the Arts.

To find out more and to register for your cat to take part in the project, check: www.discoverycircle.org.au

Contact for interview: Philip Roetman office (08) 8302 1081 email philip.roetman@unisa.edu.au
Media contact: Will Venn office (08) 8302 0096 email will.venn@unisa.edu.au
