# Supplementary material for: The ‘Feline Five’: An exploration of personality in pet cats (Felis catus)
Source: PLoS One. 2017 Aug 23;12(8):e0183455. doi: 10.1371/journal.pone.0183455 (PMC5568325; doi:10.1371/journal.pone.0183455)
Supplement: S3 Appendix — (PDF) [file pone.0183455.s003.pdf]

S3 Appendix: Preliminary Analyses

Examining factorability of the 52 cat personality survey items (New Zealand, South Australia as separate and combined samples).

Initially, the factorability of the 52 cat personality survey items was examined using several well-known criteria. Firstly, inspection of the correlation matrix revealed reasonable factorability by numerous coefficients of .30 and above [55]. However, the suggestion of very closely linearly related items, or multicollinearlity, was detected by the Haitovsky test [78] of the determinant value of 9.801×10^{-10} as less than the necessary value of 1×10^{-5} [50, 56]. The removal of single theoretical items from pairs of items believed to be measuring the same construct was therefore explored (e.g. insecure and anxious). However, the decision was made to remain conservative and not eliminate items at this stage, as there were no obvious drawbacks to taking this action when assessing the suitability of the factors extracted from the data [56]. Secondly, the Kaiser-Meyer-Olkin (KMO) measure of sampling adequacy was .92, which is well above the minimum value of .50 [51], and Bartlett's test of sphericity [52] was significant ($p < .001$). All the KMO values (diagonals of the anti-image correlation matrix) for individual items were .82 or more, which is above the recommended value of .50 [50], thus supporting the inclusion of all the items in the analysis. Finally, the communalities were .30 or greater (Table A, column: SA/NZ PCA), which further confirmed the personality items shared some common variance with other items. Following these preliminary analyses (see Table A), the 52 cat personality survey items were subjected to Exploratory Factor Analyses (see Supporting Information S5).

References

(Note: the additional reference in Supporting Information S3 continues after number 77 in the manuscript)

78. Haitovsky Y. Multicollinearity in regression analysis: Comment. Rev Econ Stat. 1969;51: 486-489.

Table A. Communalities of the Cat Personality Survey Items for all Factor Analyses

Item	SA 4-factor PAF (52 items)	NZ 4-factor PAF (52 items)	SA/NZ PCA (52 items)	SA/NZ PAF (52 items)	SA/NZ 5-factor PAF (48 items)
Vigilant	.25	.30	.44	.28	.28
Stable	.50	.52	.60	.52	.52
Bold	.47	.51	.58	.52	.52
Clumsy	.28	.23	.49	.28	.27
Defiant	.46	.48	.53	.47	.47
Gentle	.43	.42	.59	.46	.48
Constrained	.21	.25	.50	.24	.25
Inquisitive	.51	.46	.61	.49	.51
Inventive	.47	.40	.51	.44	.44
Irritable	.43	.41	.63	.45	.45
Distractible	.28	.29	.56	.28	.29
Erratic	.48	.47	.59	.52	.51
Solitary	.26	.22	.51	.29	.27
Impulsive	.38	.32	.49	.40	.39
Quitting	.16	.16	.56	.16	.16
Independent	.15	.17	.47	.21	-
Smart	.39	.41	.53	.40	.38
Jealous	.25	.24	.51	.38	.38
Fearful of other cats	.23	.25	.61	.24	.24
Persevering	.23	.22	.48	.23	.22
Greedy	.16	.18	.47	.25	.25
Friendly to other cats	.29	.23	.64	.28	.28
Submissive	.31	.38	.56	.34	.34
Dominant	.50	.55	.67	.57	.57
Reckless	.40	.40	.48	.40	.40
Predictable	.15	.17	.55	.25	.25
Suspicious	.62	.64	.72	.62	.62
Individualistic	.09	.15	.62	.11	-
Affectionate	.30	.25	.54	.43	.43
Insecure	.65	.65	.70	.68	.68
Bullying	.48	.58	.70	.64	.65
Curious	.54	.49	.61	.52	.53
Aimless	.36	.34	.58	.35	.35
Deliberate	.31	.29	.62	.31	.31
Tense	.36	.40	.57	.38	.37
Fearful of people	.61	.60	.73	.60	.60
Cool	.23	.24	.52	.24	.24
Aggressive to people	.29	.29	.48	.31	.31
Calm	.50	.49	.62	.50	.51
Aggressive to other cats	.43	.44	.66	.50	.51
Excitable	.35	.31	.42	.35	.35
Friendly to people	.47	.47	.69	.52	.52
Playful	.43	.30	.52	.39	.40
Vocal	.10	.06	.30	.12	-
Decisive	.48	.43	.64	.48	.46
Self-assured	.51	.55	.61	.53	.52
Anxious	.61	.60	.67	.63	.63
Trusting	.62	.60	.72	.61	.61
Active	.33	.37	.54	.39	.39
Cooperative	.25	.25	.41	.27	.27
Shy	.56	.53	.60	.55	.55
Eccentric	.16	.17	.59	.17	-
