# Supplementary material for: The ‘Feline Five’: An exploration of personality in pet cats (Felis catus)
Source: PLoS One. 2017 Aug 23;12(8):e0183455. doi: 10.1371/journal.pone.0183455 (PMC5568325; doi:10.1371/journal.pone.0183455)
Supplement: S4 Appendix — (PDF) [file pone.0183455.s004.pdf]

S4 Appendix: South Australia and New Zealand as independent samples

Scree plots and parallel analysis decision tables supporting two independent principal axis exploratory factor analyses.

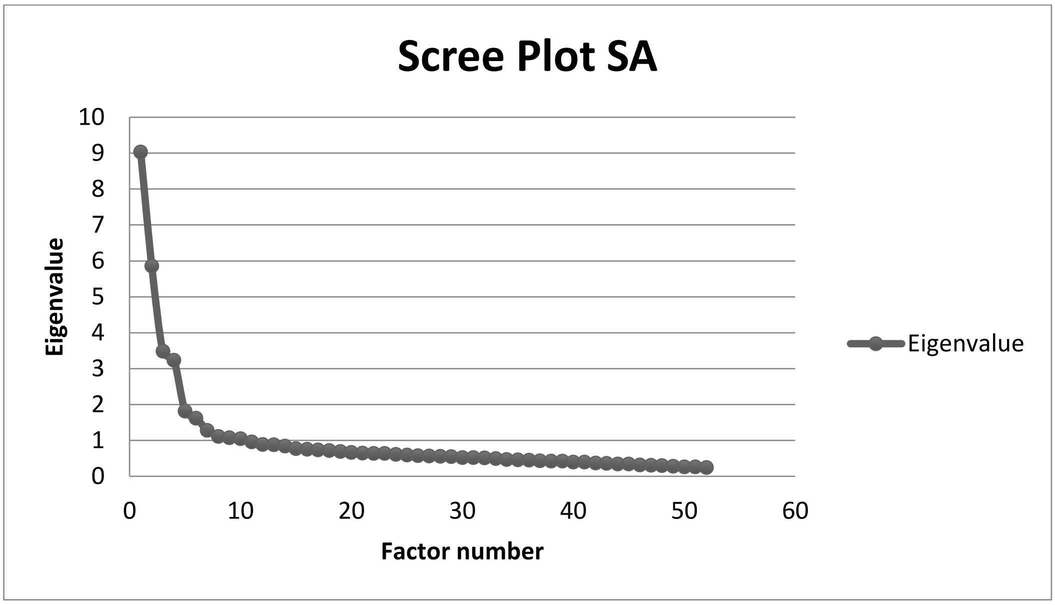

Fig A. Scree plot for the South Australian cats (n = 1,687) subjected to principal axis factor analysis.

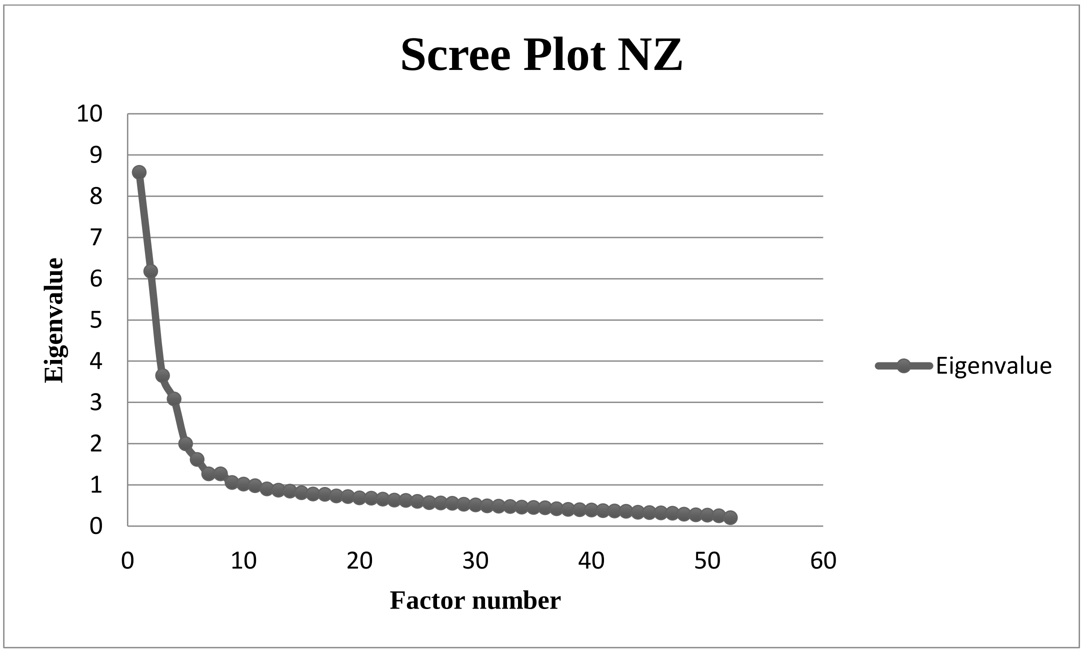

Fig B. Scree plot for the New Zealand cats (n = 1,115) subjected to principal axis factor analysis.

Table A. Comparison of Eigenvalues from the PAF (SA) and Criterion Values from Parallel Analysis

Factor number	Actual eigenvalue from PAF	Criterion value from parallel analysis	Decision
1	9.04	1.35	Accept
2	5.86	1.32	Accept
3	3.49	1.30	Accept
4	3.24	1.28	Accept
5	1.82	1.26	Accept/reject
6	1.63	1.24	Accept/reject
7	1.28	1.22	Accept/reject
8	1.12	1.21	Reject

Note. PAF = Principal Axis Factor Analysis. SA = South Australia.

Table B. Comparison of Eigenvalues from the PAF (NZ) and Criterion Values from Parallel AnalysisFactor number	Actual eigenvalue from PAF	Criterion value from parallel analysis	Decision
1	8.58	1.44	Accept
2	6.18	1.40	Accept
3	3.66	1.37	Accept
4	3.08	1.34	Accept
5	2.00	1.32	Accept/reject
6	1.62	1.30	Accept/reject
7	1.27	1.28	Accept/reject
8	1.27	1.26	Accept/reject
9	1.06	1.24	Reject

Note. PAF = Principal Axis Factor Analysis. NZ = New Zealand.
