# Supplementary material for: The ‘Feline Five’: An exploration of personality in pet cats (Felis catus)
Source: PLoS One. 2017 Aug 23;12(8):e0183455. doi: 10.1371/journal.pone.0183455 (PMC5568325; doi:10.1371/journal.pone.0183455)
Supplement: S5 Appendix — (PDF) [file pone.0183455.s005.pdf]

S5 Appendix: Exploratory factor analysis

Principal components analysis (PCA) was initially used to determine the potential number of factors within the data set. The analysis revealed ten components with eigenvalues over Kaiser's criterion of one, cumulatively explaining a variance of 56.76%. The scree plot displaying the eigenvalues associated with the components in descending order against the number of the components was ambiguous and showed inflexion points that justified retaining two, four, or five components (see Figure A). Based on Cattell's (1966) scree test [53], which is suitable for large samples [79], a five-factor principal axis factor (PAF) analysis solution on all 52 items explaining a variance of 45.03% was preferred. Throughout the process, a total of four items (independent, individualistic, eccentric and vocal) were excluded due to not contributing to a simple factor structure having not met the minimum criterion factor loading of .30 or above (also recommended for large samples [55]; see Table A, un-rotated principal axis factor analysis loadings on 52 cat personality traits). Item removal was further supported by low corresponding communalities, or the limited proportions of each items' variance that could be explained by the factors [55]. A final PAF analysis was conducted on the 48-item cat personality dataset with five factors cumulatively explaining a variance of 47.43%.

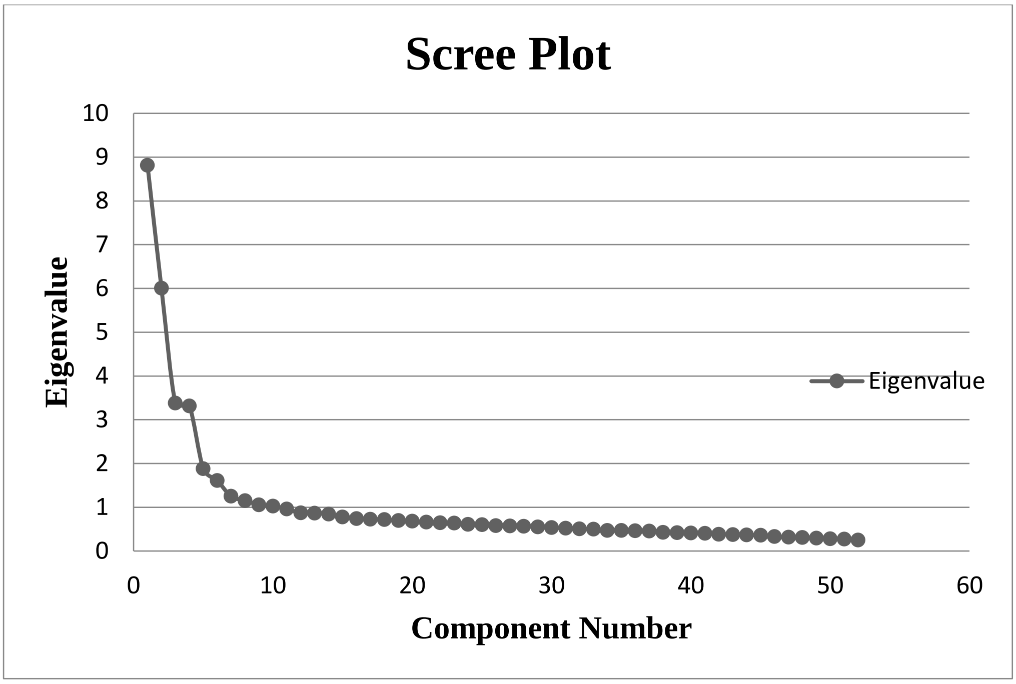

Figure A. Scree plot of the eigenvalues for the factors that emerged from the principal components analysis of the 52 variables (items/traits) measuring personality in cats.

The Oblique (Direct Oblimin) Rotated Five-Factor Solution for the ‘Feline Five’ (Table B) and the Correlations between the Factors (Factor Correlation Matrix, Table C) are provided for the combined South Australia and New Zealand sample.

Table A. Principal Axis Unrotated Five-Factor Matrix Loadings on the 52 Cat Personality Survey Items

Item	Factor 1	Factor 2	Factor 3	Factor 4	Factor 5
Insecure	**-.74**	-.10	.28	.14	.14
Anxious	**-.74**	-.06	.21	.12	.13
Trusting	**.69**	.03	**-.31**	.20	.03
Fearful of people	**.67**	-.05	**.37**	-.11	.01
Shy	**-.67**	-.18	.24	-.05	.07
Stable	**.67**	-.12	-.12	-.20	-.07
Suspicious	**-.66**	-.03	**.38**	-.19	.01
Calm	**.65**	-.10	-.24	-.12	-.09
Self-assured	**.64**	.24	.11	-.22	-.01
Tense	**-.59**	.08	.11	.05	.05
Friendly to people	**.58**	-.01	-.22	**.30**	.20
Bold	**.53**	**.46**	-.02	.03	-.17
Inquisitive	**.46**	.29	**.33**	.28	-.03
Fearful of other cats	**-.43**	-.17	.13	.07	.04
Curious	**.43**	**.32**	**.40**	.27	-.05
Affectionate	**.43**	-.18	.04	.28	**.36**
Solitary	**-.41**	.06	.01	-.27	-.22
Cool	**.41**	-.01	-.14	-.22	-.09
Inventive	**.39**	**.33**	**.38**	.20	.01
Cooperative	**.33**	**-.31**	.17	.10	.14
Quitting	**-.31**	.04	-.22	.12	-.03
Bullying	-.08	**.67**	-.21	-.22	**.31**
Defiant	-.04	**.66**	-.11	-.13	.03
Dominant	.09	**.65**	-.17	-.26	.21
Aggressive to other cats	-.16	**.57**	-.15	-.26	.24
Reckless	-.06	**.55**	-.12	.28	-.06
Irritable	**-.35**	**.51**	-.15	-.15	-.16
Erratic	**-.41**	**.48**	-.14	.25	-.19
Impulsive	-.13	**.47**	.02	**.35**	-.20
Gentle	**.41**	**-.47**	.07	.17	.20
Jealous	-.18	**.45**	-.11	-.07	**.35**
Submissive	-.17	**-.43**	.10	**.33**	-.07
Aggressive to people	**-.30**	**.43**	-.10	-.06	-.15
Constrained	.02	**-.39**	-.04	-.25	.15
Persevering	.22	**.35**	.23	.01	.06
Greedy	-.09	**.35**	-.20	.05	.28
Predictable	.08	**-.31**	.11	-.23	.29
Individualistic	-.02	.29	.14	.09	.03
Eccentric	-.11	.29	.04	.26	.08
Smart	**.32**	.19	**.46**	-.20	-.05
Vigilant	.03	.25	**.46**	.01	-.08
Decisive	**.38**	.26	**.44**	-.24	.13
Active	.29	.27	**.41**	.18	-.20
Deliberate	.25	.16	**.36**	-.27	.13
Aimless	**-.31**	.07	**-.36**	**.34**	-.03
Playful	**.34**	.12	.28	**.41**	.11
Distractible	-.22	.23	-.14	**.40**	-.06
Clumsy	-.20	-.04	-.29	**.36**	.15
Excitable	**-.33**	.28	.15	**.35**	.12
Friendly to other cats	**.32**	-.21	.05	**.34**	-.12
Independent	.18	.22	.07	-.28	-.21
Vocal	.12	.15	.13	.18	.20

Note. Salient factor loadings (≥ .30) are in boldface.

Table B. Combined South Australian and New Zealand Pet Cat Personality Factor Structures

(*n* = 2,802)

	Oblique (Direct Oblimin Rotated PAF Analysis Loadings				
Item	Neuroticism	Dominance	Extraversion	Impulsiveness	Agreeableness
Insecure	**-.84**	-.01	-.03	-.02	.07
Anxious	**-.78**	.03	-.07	-.01	.03
Fearful of people	**-.73**	-.04	.15	-.08	-.20
Suspicious	**-.72**	.00	.18	-.12	-.24
Shy	**-.70**	-.06	-.02	-.14	-.10
Calm	**.69**	-.08	-.04	-.10	.00
Trusting	**.68**	.01	-.11	.10	**.30**
Stable	**.64**	-.09	.08	-.18	-.01
Tense	**-.55**	.09	-.06	.06	-.07
Bold	**.52**	.12	.29	.33	-.03
Self-assured	**.49**	.12	**.40**	-.07	-.01
Cool	**.47**	.01	.03	-.12	-.11
Fearful of other cats	**-.46**	-.10	-.08	-.06	.01
Excitable	**-.43**	.15	.06	**.30**	.22
Bullying	.05	**.80**	.02	.01	.00
Dominant	.20	**.70**	.12	.02	-.06
Aggressive to other cats	-.03	**.70**	.03	-.02	-.08
Jealous	-.14	**.62**	-.02	-.03	.12
Defiant	.11	**.52**	.12	.24	-.16
Greedy	-.02	**.48**	-.13	.05	.17
Submissive	-.26	**-.45**	-.15	.05	.17
Friendly to other cats	.19	**-.37**	.01	.16	.23
Decisive	.06	.16	**.62**	-.17	.03
Smart	.03	.00	**.61**	-.07	-.04
Curious	.08	-.05	**.55**	**.30**	.27
Inventive	.05	.02	**.53**	.22	.25
Active	.03	-.17	**.52**	**.33**	.06
Vigilant	-.22	-.03	**.51**	.14	-.02
Deliberate	-.01	.15	**.50**	-.24	.00
Inquisitive	.13	-.04	**.49**	.28	**.30**
Aimless	-.06	.05	**-.47**	**.33**	.08
Clumsy	-.09	.06	**-.41**	.15	.25
Persevering	.04	.18	**.38**	.10	.08
Quitting	-.11	.06	**-.31**	.15	-.04
Impulsive	-.06	.05	.08	**.60**	-.01
Erratic	-.15	.16	-.14	**.57**	-.17
Predictable	-.09	.05	.05	**-.51**	.14
Reckless	.05	.26	.00	**.50**	.04
Constrained	-.01	-.05	-.12	**-.47**	.01
Distractible	-.11	.04	-.20	**.44**	.12
Aggressive to people	-.07	.23	-.02	**.31**	**-.30**
Affectionate	.10	.03	.03	-.19	**.61**
Friendly to people	**.46**	.07	-.10	.03	**.49**
Playful	-.02	-.07	**.32**	.20	**.47**
Gentle	.12	-.25	-.02	**-.31**	**.46**
Solitary	-.21	.00	-.05	.02	**-.43**
Irritable	-.05	.32	-.04	**.30**	**-.40**
Cooperative	.05	-.20	.12	-.23	**.32**

Note. PAF = Principal Axis Factor. Salient factor loadings (≥ .30) are in boldface.

Table C. Factor Correlation Matrix

Factor	Neuroticism	Dominance	Extraversion	Impulsiveness	Agreeableness
Neuroticism	1.00	-.03	.25	-.11	.24
Dominance	-.03	1.00	.05	.28	-.20
Extraversion	.25	.05	1.00	.01	.07
Impulsiveness	-.11	.28	.01	1.00	.08
Agreeableness	.24	-.20	.07	.08	1.00

References

(Note: the additional reference in Supporting Information S5 continues after number 78 in Supporting Information S3)

79. Stevens JP. Applied multivariate statistics for the social sciences. 5th ed. New York: Routledge; 2012.
