# Supplementary material for: The ‘Feline Five’: An exploration of personality in pet cats (Felis catus)
Source: PLoS One. 2017 Aug 23;12(8):e0183455. doi: 10.1371/journal.pone.0183455 (PMC5568325; doi:10.1371/journal.pone.0183455)
Supplement: S6 Appendix — (PDF) [file pone.0183455.s006.pdf]

S6 Appendix: Comparison of Factor Labels used in other animal and particularly felid studies compared with the current study.

Table A. Cross-Species Similarities from Previous Personality Research to Current Pet Cat Personality Research

Study	Species (& environment)	Personality factor label	Traits
Current study	***Domestic cat (private homes)***	***Neuroticism***	***+Insecure +Anxious +Fearful of people +Suspicious +Shy +Tense +Fearful of other cats +Excitable –Trusting –Calm –Stable –Self-assured –Bold –Cool***
[37]	Domestic cat (shelters)	Neuroticism	+Anxious +Insecure +Tense +Suspicious +Fearful of people –Stable –Trusting –Cool –Calm –Self-assured
[37]	Five felids	Neuroticism	+Fearful of people +Suspicious +Insecure +Tense +Anxious +Fearful of conspecifics -Trusting –Cool –Calm–Self-assured –Stable
[47]	Snow leopard (zoos)	Timid-anxious	+Anxious +Insecure +Tense +Excitable
[20]	Cheetah (zoos)	Tense-fearful	+Fearful of conspecifics +Fearful of people +Insecure +Tense -Calm –Self-assured
Current study	***Domestic cat (private homes)***	***Extraversion***	***+Decisive +Smart +Curious +Inventive +Active +Inquisitive +Vigilant +Deliberate +Persevering –Aimless –Clumsy –Quitting***
[47]	Snow leopard (zoos)	Active/vigilant	+Active +Vigilant
[66]	Bengal tiger (zoo)	Extraversion	+Vigilant +Active
[25]	Domestic cat (private homes)	Active	+Curious +Active +Vigilant
[59]	Orangutan (zoos)	Extraversion	+Active +Curious +Inquisitive +Inventive
[20]	Cheetah (zoos)	Excitable-vocal	+Active +Smart
[19]	Scottish wildcat (zoos)	Self-control	+Decisive +Persevering +Smart –Aimless –Quitting
Current study	***Domestic cat (private homes)***	***Dominance***	***+Bullying +Dominant +Aggressive to other cats +Jealous +Defiant +Greedy –Submissive –Friendly to other cats***
[37]	Domestic cat (shelters)	Dominance	+Aggressive to conspecifics +Bullying +Dominant +Jealous +Stingy –Submissive –Friendly to conspecifics
[37]	Five felids	Dominance	+Dominant +Aggressive to conspecifics +Stingy +Bullying +Jealous
[19]	Scottish wildcat (zoos)	Dominance	+Dominant +Bullying +Stingy +Aggressive to conspecifics +Jealous –Friendly to conspecifics
Current study	***Domestic cat (private homes)***	***Impulsiveness***	***+Impulsive +Erratic +Reckless +Distractible –Predictable –Constrained***
[37]	Domestic cat (shelters)	Impulsiveness	-Constrained +Impulsive +Distractible +Reckless +Erratic –Predictable
Current study	***Domestic cat (private homes)***	***Agreeableness***	***+Affectionate +Friendly to people +Gentle +Playful +Cooperative –Solitary –Irritable –Aggressive to people***
[19]	Scottish wildcat (zoos)	Agreeableness	+Cooperative +Friendly to people +Playful
[59]	Orangutan (zoos)	Agreeableness	+Affectionate +Friendly +Gentle

Note. Five felids = domestic cat (shelters), clouded leopard (zoos), Scottish wildcat (zoos), snow leopard (zoos), African lion (zoos). Conspecifics = the same species. Greedy = 'Stingy' in other research.
